# Monitoring the Growth and Habitat Shifts of Epiphyllous Liverworts in Subtropical Forests of China

**DOI:** 10.1002/ece3.71442

**Published:** 2025-05-12

**Authors:** Zun Dai, Yu‐Ting Yang, Yi‐Ran Wang, Xue Yao, Li‐Jie Zhuang, Mao‐Jie Zhu, Jian Zhang, Jian Wang

**Affiliations:** ^1^ Bryology Laboratory School of Life Sciences, Zhejiang Zhoushan Island Ecosystem Observation and Research Station, School of Ecological and Environmental Sciences, East China Normal University Shanghai China; ^2^ School of Life Sciences, Sun Yat‐Sen University Guangzhou China; ^3^ Shanghai Institute of Eco‐Chongming (SIEC) Shanghai China

**Keywords:** bryophytes, climate change, dispersal capacities, epiphyllous liverworts, habitat preference, indicator species

## Abstract

Understanding the extent to which species can adjust their ranges in response to climate change and track areas of suitable climatic conditions is vital for conservation efforts. Nonetheless, the observed changes in species distribution may also result from inadequate field data. This is particularly relevant for epiphyllous liverworts, which exhibit a poikilohydric lifestyle that makes them more vulnerable to climatic fluctuations than many other higher plants. Furthermore, their small plant size increases the chances of under‐detection in epiphyllous liverworts compared to other plant groups. To enhance our understanding of how species distribution is influenced by climate change, establishing long‐term monitoring plots is essential. In this study, we utilize the BEST platform (Biodiversity along Elevational Gradients: Shifts and Transitions) to furnish empirical evidence regarding the habitat shifts of epiphyllous liverworts along the elevational gradient of Mt. Tianmu. To identify the specific microclimatic conditions vital for the growth and development of epiphyllous liverworts, we conducted a transplant experiment. Our systematic observations from the permanent monitoring plots (2018–2022) led to the discovery of a new population of epiphyllous liverworts located at an elevation of 1130 m. By analyzing in situ microclimatic data on air temperature and moisture, collected consistently over 5 years (2017–2022), we characterized the mean, minimum, and variability of the microclimatic conditions essential for epiphyllous liverwort growth. Additionally, results from elevation transplantation experiments underscore the importance of incorporating dispersal constraints when modeling the species distribution of epiphyllous liverworts for precise predictive outcomes. Our results highlight the importance of long‐term monitoring permanent plots in predicting the effects of global changes on species habitat shifts, and underscore the necessity for comprehensive investigations of the distribution of epiphyllous liverworts at the northern boundary of subtropical evergreen broad‐leaved forests in China.

## Graphical abstract

Our observations from the permanent monitoring plots indicate a rapid expansion in the distribution range of epiphyllous liverworts under the influence of climate change. We have identified the specific microclimate conditions necessary for the growth of epiphyllous liverworts. The results from elevation transplantation experiments emphasize the significance of considering dispersal limitations when modeling the species distribution of epiphyllous liverworts for accurate predictive outcomes.

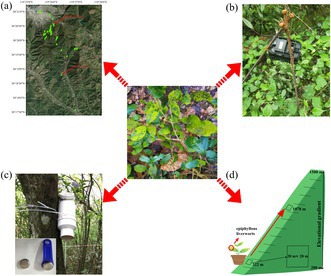

## 1 Introduction

In the context of climate change, understanding the current and future distribution of species is crucial for their conservation (Guisan et al. 2013; Sutherland et al. 2013). Climate, particularly temperature, has long been recognized as a key factor influencing the geographic ranges of species on a large scale (Gaston 2003). There is abundant evidence suggesting that species have moved towards higher latitudes and elevations due to climate change (Walther et al. 2002 and references therein; Lovejoy and Hannah 2005), although a direct correlation between temperature change and the observed range shifts could usually not be shown (cf. Chen et al. 2011). Also, it remained uncertain whether the observed shifts in elevational range were driven by natural seasonal variability or by a potential bias in data collection resulting from the lack of repeated observations over time (Maicher et al. 2020). The latter issue is especially relevant to plants, which are often incompletely recorded (Chen et al. 2013).

Numerous studies have demonstrated that body size and extent of geographic range significantly influence the probability of species being gathered by collectors (e.g., Collen et al. 2004; Essl et al. 2013; Colli et al. 2016), and that organisms with large geographic distributions and considerable body size are more likely to be recorded than those with smaller ranges and body sizes (Collen et al. 2004). Among the land plants, due to their small body size and specific requirements for microhabitats, bryophytes tend to thrive in microhabitats. As a result, the issue of imperfect recording is particularly relevant to the bryophytes due to their relatively small body size and their preference for microhabitats (Vanderpoorten and Goffinet 2009).

This paper focuses on epiphyllous liverworts. Epiphyllous liverworts are a highly specialized group of bryophytes that typically grow on living leaves in the shaded understory of humid tropical and subtropical forests. Due to their specific microhabitat requirements, they are particularly sensitive to changes in moisture and temperature and are considered highly sensitive bioindicators of climate change (e.g., Pócs 1996; Zartman 2003; Malombe et al. 2016; Jiang et al. 2014, 2018).

In China, the spatial distribution of epiphyllous liverworts may vary over time because of climate change (Jiang et al. 2018). Since Chen and Wu (1964), epiphyllous species were considered widely distributed in tropical rainforests and subtropical evergreen broad‐leaved forests up to 30° north latitude. Subsequently, their distribution range has gradually extended northwards beyond 30°N as evidenced by studies in Hubei and Zhejiang Provinces (Peng et al. 2002; Tang et al. 2018; Du et al. 2020). Nevertheless, solid empirical evidence is necessary to support the conclusion that the new records were the result of a recent northward migration and not due to incomplete previous inventorying (Frahm and Klaus 2001).

Climatic factors, including mean annual temperature, mean annual precipitation, minimum temperature of the coldest month, precipitation during the driest month, and seasonality of temperature and precipitation, are considered drivers of plant and animal distributions (Kooyman et al. 2012; Qian et al. 2019). Jiang et al. (2014) found that temperature seasonality, coldest quarter temperature, and precipitation during the wettest month and quarter might be key factors influencing the distribution range of epiphyllous liverworts in China. Nevertheless, in order to more precisely delineate the microclimate factors influencing the distribution of epiphyllous liverworts, it is essential to conduct long‐term monitoring of their growth environments (De Frenne et al. 2021).

The establishment of permanent monitoring plots is considered a suitable approach to the generation of pertinent data on range shifts of species (Magurran et al. 2010). In order to study the dynamics of biodiversity in response to climate shifts across various taxa (vascular plants, bryophytes, soil microbes, soil fauna, birds), a long‐term research initiative known as BEST (Biodiversity along Elevational Gradients: Shifts and Transitions) was established in 2017 (https://best‐mountains.org/). This project, which involved a collaboration of more than 20 research teams, resulted in the creation of 243 permanent plots positioned at different elevations across 12 mountain areas in between 19.07° N and 43.37° N, and extending from 700 to 2345 m. Utilizing the BEST platform, we are able to carry out long‐term observations on the possible distribution changes of epiphyllous liverworts in response to climate change. The monitoring, which focuses on changes along both latitude and elevation gradients, will allow us to gain more precise insights into the effects of climate‐related factors on these organisms over time.

Utilizing the BEST platform, the specific aims of our study include: (1) gathering empirical data regarding distribution changes of epiphyllous liverworts along the elevational gradient on Mt. Tianmu, Zhejiang Province (Tang et al. 2018); and (2) determining the specific microclimate conditions that are essential for the successful growth and development of these highly specialized organisms, thereby deepening our understanding of their ecological requirements.

## 2 Methods

### 2.1 Study Area

This study was carried out in the Tianmushan National Nature Reserve (TNNR) in Mt. Tianmu, Zhejiang Province, China (119°23′–119°28′ E, 30°18′–30°24′ N), at the northern boundary of the mid‐subtropical zone and encompassing an area of 4284 hm^2^, between 200 and 1506 m. The area is characterized by a humid monsoon climate, with annual temperature fluctuations from 8.8°C to 14.8°C and an average yearly rainfall between 1390 and 1870 mm. The topography and climatic conditions lead to the presence of three distinct vegetation zones: (1) evergreen broad‐leaved forest zone (below 950 m), (2) mixed forest zone of evergreen and deciduous broad‐leaved tree species (950–1200 m), and (3) the deciduous broad‐leaved forest zone (1200–1506 m) (Shang et al. 2018). In 2017, we found five epiphyllous liverwort species newly from the lowland area of Mt. Tianmu, and proposed that the establishment of the epiphyllous species in the region was the result of a recent event influenced by climate warming (Tang et al. 2018).

### 2.2 Data Sampling

**FIGURE 1:**
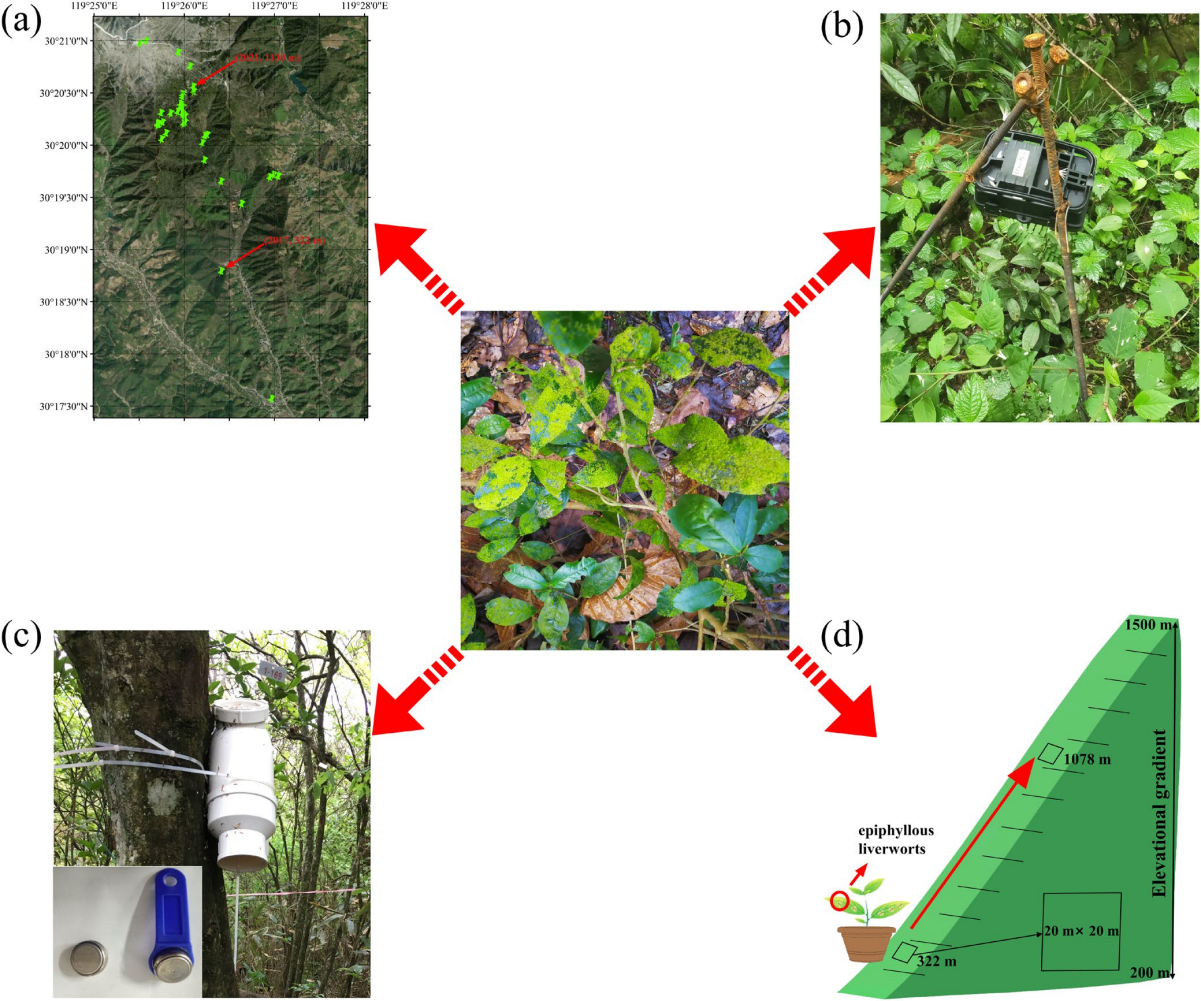
Long‐term monitoring distribution change of epiphyllous liverworts by permanent plots in Mt. Tianmu, Zhejiang Province, China. (a) thirty‐six 20 m × 20 m plots were set up in 2017–2018 along an elevational gradient from 270 m to 1470 m above sea level in this mountain, at intervals of ~100 m in elevation (arrows indicate two sites of epiphyllous liverworts found in Mt. Tianmu); (b) phenological camera; (c) air temperature and relative humidity were recorded 1 m above ground using a data logger (iButtons DS1923#F5; Maxim Integrated Products, USA) placed in a solar radiation shield; (d) transplantation experiment and method of epiphyllous liverworts.

To monitor the distribution of epiphyllous liverworts, 36 plots measuring 20 m × 20 m were established during 2017 and 2018 between 270 and 1470 m, at elevational intervals of ca. 100 m (Figure 1a). In addition, six phenological cameras were positioned along the stream at 321 m elevation, where epiphyllous liverworts were found in 2017. The cameras focus on specific target leaves that did not host epiphyllous liverworts (Figure 1b). Each camera was programmed to capture and record data at two‐hour intervals. Subsequently, we will determine the duration required for epiphyllous liverworts to establish themselves on a bare leaf surface by screening the photographs taken by these cameras.

To assess water availability and air temperature required for the growth and distribution of epiphyllous liverworts, we gathered microclimate data across different elevational gradients (270–1470 m). The calculations for mean, minimum, as well as seasonality of air temperature and moisture were performed based on the definitions provided by WorldClim (Fick and Hijmans 2017). Average air temperature and relative humidity values from the years 2017 to 2022 were included as microclimatic variables. In each plot, data on air temperature and moisture were collected utilizing a single iButton DS1923‐F5 datalogger (Maxim Integrated Products, USA), programmed to sample at 2‐h intervals. The datalogger was mounted on the northern side of a tree trunk, at 1 m above ground level. To shield the sensors from direct sunlight, the datalogger was encased in a white PVC shield (Figure 1c).

To better understand the microclimatic conditions necessary for the growth and development of epiphyllous liverworts, a comprehensive field experiment was conducted (2019–2023) (Figure 1d). In this study, ten *Camellia sinensis* plants were carefully cultivated in flower pots at the site (321 m) where epiphyllous liverworts were first discovered. After a six‐month period, a total of 190 leaves were observed to be colonized by epiphyllous liverworts (Figure 4b). The *C. sinensis* plants holding epiphyllous liverworts were subsequently moved from 321 to 1077 m, where *C. sinensis* plants were thriving naturally in great abundance.

To assess the growth and spread of epiphyllous liverworts at the higher‐elevation site (1077 m), leaves inhabited by epiphyllous liverworts were marked (Figure 4d) and subsequently monitored every 8 months. In addition, the epiphyllous species growing on the transplanted host plants were sampled randomly, and their chloroplasts were scrutinized with the aid of a light microscope in order to assess the overall health and vitality of the epiphyllous species following transplantation.

## 3 Results

**FIGURE 2:**
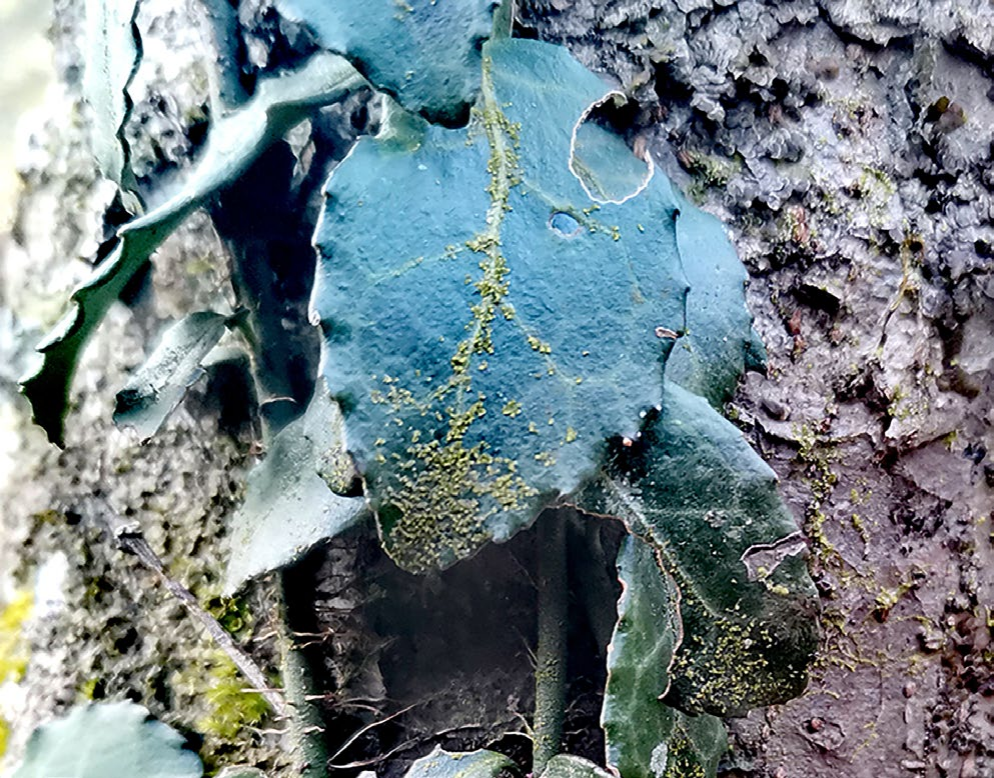
A new occurrence of an epiphyllous species (*Cololejeunea tianmuensis*) found at a plot situated at an elevation of 1130 m in Mt. Tianmu in 2022.

**FIGURE 3:**
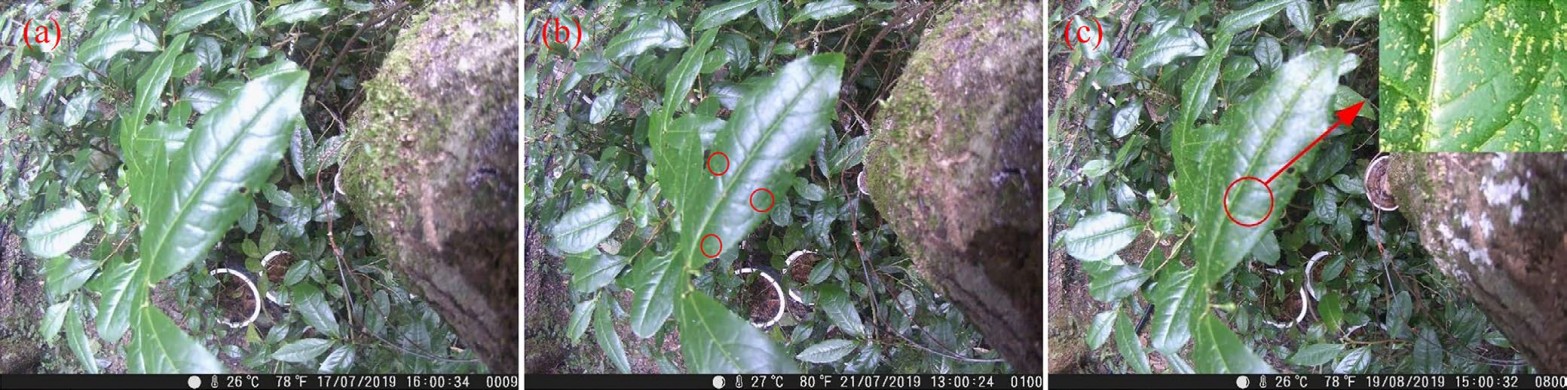
Colonization of epiphyllous liverworts recorded by phenological camera in Mt. Tianmu. (a) No colonization of epiphyllous liverworts; (b) initial sighting of colonization of epiphyllous liverworts; (c) a mature population of epiphyllous liverworts (circles indicate the presence of epiphyllous liverworts on the leaf).

After a four‐year monitoring of the distribution of epiphyllous liverworts on Mt. Tianmu (2018–2022), the new occurrence of one epiphyllous species, *Cololejeunea tianmuensis*, was detected in a plot situated at 1130 m (Figure 2). The data captured by only one of the six cameras at 321 m, furthermore, indicated that the new colonization of epiphyllous liverworts on unoccupied leaves took approximately six months (Figure 3).

**FIGURE 4:**
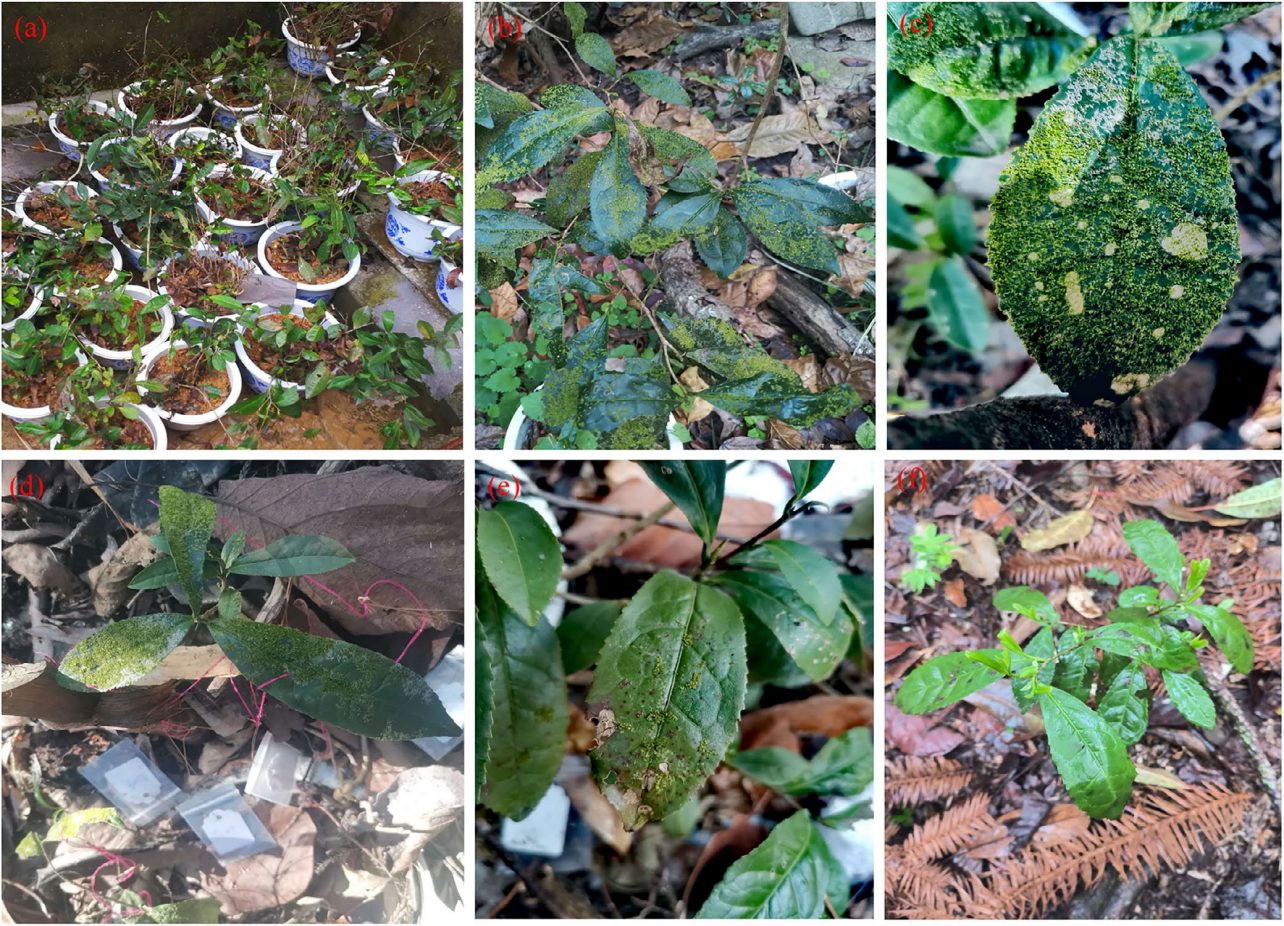
The process and results of elevation transplantation of epiphyllous liverworts in Mt. Tianmu. (a) The *Camellia sinensis* plants cultivating in flower pots at the original site (321 m) where epiphyllous liverworts were first discovered; (b) the leaves of *C. sinensis* that have been colonized by liverworts; (c) ~ (f) shows the health status of the transplanted host plants and epiphyllous liverworts at high elevation (1077 m) over time; (c) August 2020; (d) April 2021; (e) January 2022; (f) September 2023.

The transplanted host plants along with the epiphyllous liverworts maintained in good health from August 2020 to January 2022 (Figure 4). Nevertheless, the number of leaves inhabited by epiphyllous liverworts gradually declined as older leaves were falling off. By September 20, 2023, no epiphyllous liverworts were observed on the transplanted host plants (Table S1). Additionally, throughout the three‐year transplantation experiment, no new leaves of the transplanted host plants were colonized by epiphyllous liverworts.

The field study indicated that epiphyllous liverworts were capable of thriving at three distinct elevations, 321, 1077, and 1130 m. Moreover, the five‐year monitoring of microclimatic conditions indicated that the growth and distribution of epiphyllous liverworts correlated with the following parameters (Table S2): (1) mean annual temperature higher than 11.5°C, (2) mean temperature of coldest quarter higher than 1.6°C, (3) temperature seasonality lower than 7.6°C, (4) mean annual moisture higher than 86.2%, (5) mean moisture of coldest quarter higher than 83.8%, and (6) moisture seasonality lower than 0.06.

## 4 Discussion

Our results show that monitoring of permanent plots can yield valuable new, empirical data regarding the distribution changes of epiphyllous liverworts along elevational gradients. Notably, the results identified a new location of epiphyllous liverworts at an elevation of 1130 m on Mt. Tianmu in 2022, 800 m higher than was known in the area. The finding suggests that epiphyllous liverworts possess a remarkable capacity to track suitable habitats in response to specific climatic conditions. While the mean annual temperature of this new site is considerably less than that of the location at a lower elevation (321 m), the variation in mean annual moisture levels between the two sites is not significant (Table S2). It is important to clarify that the presence of epiphyllous species at this new location cannot be attributed to a range shift from lower elevations; rather, it indicates a shift in habitat from the adjacent tree trunks. Through a thorough comparison of the epiphyllous species found in this area with their neighboring epiphytic species at the lower elevation site (321 m), we have discovered that the majority of these epiphyllous species come from the nearby tree trunks (see Table S3 for details). Additionally, the epiphyllous species observed at the higher elevation site (1130 m) also come from the adjacent tree trunks (illustrated in Figure 2). Consequently, we tentatively propose that when the temperature and humidity levels in a certain area meet the conditions necessary for the growth of epiphyllous liverworts, there is a potential for certain species, particularly Lejeuneaceae species that develop on tree trunks, to successfully disperse and establish themselves on the lower leaves. Further research and evidence will be essential to support this speculation.

Apart from enhancing our comprehension of habitat shifts for epiphyllous liverworts along elevational gradients, the long‐term observation of permanent plots may contribute to a better understanding of the essential environmental factors that influence the distribution of these liverworts. Our long‐term monitoring of microclimates along the elevational gradient on Mt. Tianmu yielded valuable insights, including the mean, minimum, and variability of microclimatic data pertinent to the growth and distribution of epiphyllous liverworts in Mt. Tianmu. Our results provide partial support for the distribution predictions made regarding epiphyllous liverworts in China, as informed by macroclimatic variables (Jiang et al. 2014). Their model suggests that epiphyllous liverworts are likely found in regions where temperature seasonality is below 7°C, the annual temperature range is beneath 28°C, and the coldest quarter's temperature exceeds 5°C. The microclimatic measurements taken along the elevation gradient indicate that the actual conditions under which epiphyllous liverworts thrive in Mt. Tianmu are below these specified thresholds. This observation is understandable, as forest organisms residing beneath or within the tree canopy experience markedly different climatic conditions compared to those outside forest environments (De Frenne et al. 2019). Specifically, temperature extremes are often more moderated within forests in contrast to open habitats, resulting in cooler maximum temperatures below the canopy, warmer minimum temperatures, and reduced seasonal and interannual variability (Ewers and Banks‐Leite 2013; De Frenne et al. 2019). Therefore, it is crucial to acquire climatic data at a fine scale when investigating the factors influencing the distribution of epiphyllous liverworts in China.

In light of the growing body of evidence that underscores the necessity for a shift towards finer scales in biodiversity studies, it becomes imperative to account for the environmental conditions, particularly the climatic factors, that organisms actually encounter in their habitats (Lembrechts 2023). The findings presented in this study offer an initial insight into how epiphyllous liverworts respond to climate change, drawing from long‐term monitoring data. Nonetheless, the microclimate experienced by these organisms is contingent on their size; microscale processes in organisms measuring just a few millimeters may transpire at spatial scales below 10 cm (Pincebourde and Woods 2020). Research has shown that there can be substantial temperature variations, with reported differences of up to 10°C between the bottom and the edges of ridges on rugged surfaces (Nicolai 1986). In such scenarios, even localized microclimates, like those examined in this study, may not accurately reflect the actual climatic conditions faced by epiphyllous liverworts. To further detail the climate conditions that these liverworts truly experience, it would be necessary to transition from micro‐to nano‐climates, which would enhance our understanding of the niches occupied by epiphyllous liverworts and therefore refine our evaluation of their distribution changes in response to climate change.

Our transplant experiment also revealed that while epiphyllous liverworts thrive at high elevations (1077 m plot), their ability to disperse to new leaves is limited. In fact, when the microclimate is favorable, epiphyllous liverworts can rapidly colonize a vacant leaf. Data obtained from phenological cameras revealed that the first colonization of epiphyllous liverworts on an empty leaf occurs within a mere six months. Therefore, it is likely that the lack of appropriate dispersal conditions (such as dispersal pathways) hindered the expansion and establishment of epiphyllous liverworts on new leaves at the high altitudes of Mt. Tianmu. This underscores the necessity of considering dispersal constraints when forecasting the future distribution ranges of species, in order to enhance predictive precision (Briscoe et al. 2019).

If bryophytes are indeed anticipated to lag behind the pace of future climate change, as suggested by Zanatta et al. (2020), the observed capacity of epiphyte bryophytes to shift their ranges more rapidly compared to other habitat preferences (Dai et al. 2023) suggests that this specific group may serve as effective indicators for climate change impacts on other groups. In contrast to epiphytic bryophytes, the leaves occupied by liverworts (namely epiphyllous liverworts) are particularly appealing to casual collectors, which results in them being more readily noticed and collected by individuals lacking specialization (Gradstein et al. 1996; Yao et al. 2023). This noteworthy aspect of epiphyllous liverworts amplifies their potential as effective indicators of climate change specifically within subtropical forest ecosystems in China. Nonetheless, employing the distribution changes of epiphyllous liverworts over time as indicators of climate change requires accurate and geographically specific data regarding their current distributions, with particular attention to alterations at their northernmost limits in China. Acknowledging the crucial importance of long‐term monitoring through permanent plots, such as the BEST platform, in predicting the effects of global alterations on species range shifts, we recommend a thorough investigation and sustained monitoring of the distribution of epiphyllous liverworts along the northern edge of subtropical evergreen broad‐leaved forests in China.

## Author Contributions

**Zun Dai:** data curation (lead), investigation (supporting), methodology (lead), visualization (lead), writing – original draft (supporting), writing – review and editing (equal). **Yu‐Ting Yang:** methodology (equal), visualization (equal), writing – original draft (supporting), writing – review and editing (equal). **Yi‐Ran Wang:** writing – original draft (supporting), writing – review and editing (equal). **Xue Yao:** data curation (supporting), investigation (supporting). **Li‐Jie Zhuang:** data curation (supporting), investigation (supporting). **Mao‐Jie Zhu:** data curation (supporting), investigation (supporting). **Jian Zhang:** conceptualization (lead), data curation (supporting), funding acquisition (lead), writing – original draft (lead), writing – review and editing (equal). **Jian Wang:** conceptualization (lead), investigation (lead), methodology (supporting), supervision (lead), writing – original draft (lead), writing – review and editing (equal).

## Conflicts of Interest

The authors declare no conflicts of interest.

## Supporting information


Table S1‐S3.

## Data Availability

Data used in the analyses of this study are publicly accessible at https://doi.org/10.5061/dryad.ksn02v7ck.
