# Supplementary material for: Monitoring the Growth and Habitat Shifts of Epiphyllous Liverworts in Subtropical Forests of China
Source: Ecol Evol. 2025 May 12;15(5):e71442. doi: 10.1002/ece3.71442 (PMC12068890; doi:10.1002/ece3.71442)
Supplement: Supplementary file 1 — Table S1‐S3. [file ECE3-15-e71442-s001.docx]

TABLE S1 Monitoring the fluctuations in the number of host leaves that host epiphyllous liverworts over time. (a) The position of the ten flower pots at a plot of high elevation (1077 m); (b) changes of the number of leaves inhabited by the epiphyllous liverworts in the ten flower pots over time.


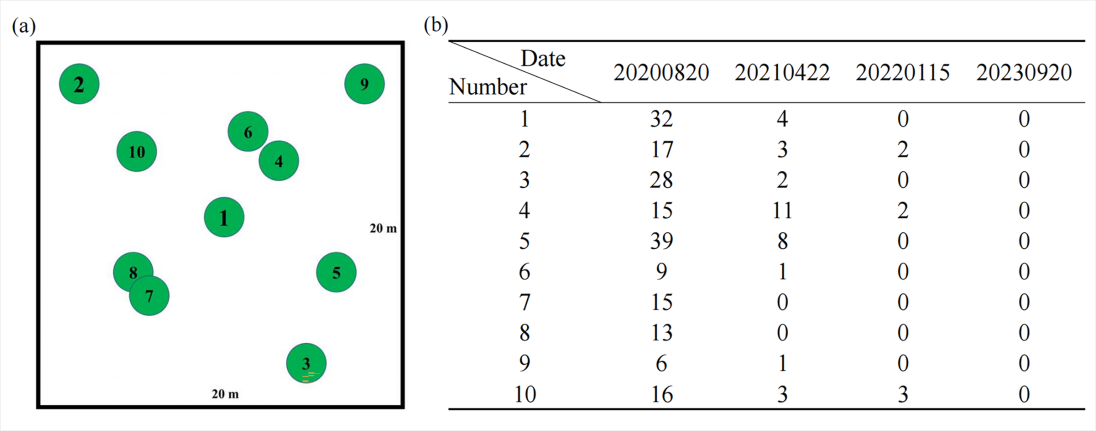


TABLE S2 The five-year microclimate data (2017–2022) of the first occurrence site (321 m), elevation transplantation site (1,077 m) and the new occurrence site (1,130 m) of epiphyllous liverworts in Mt. Tianmu, Airtemp_bio1: Mean annual temperature, Airtemp_bio4: Temperature seasonality (standard deviation *100), Airtemp_bio11: Mean temperature of coldest quarter (December, January and February), AirMois_bio1: Mean annual moisture, AirMois_bio4: Moisture seasonality (coefficient of variation), AirMois_bio11: Mean moisture of coldest quarter (December, January and February).

| Site | Elevation/m | Airtemp_bio1 | Airtemp_bio4 | Airtemp_bio11 | AirMois_bio1 | AirMois_bio4 | AirMois_bio11 |
| --- | --- | --- | --- | --- | --- | --- | --- |
| 1 | 321 | 15.3448 | 737.3005 | 5.6893 | 91.3334 | 0.0392 | 90.6354 |
| 2 | 1077 | 12.1992 | 755.9367 | 2.0881 | 86.2005 | 0.0642 | 83.7546 |
| 3 | 1130 | 11.5336 | 751.6307 | 1.5707 | 90.0918 | 0.0592 | 86.2174 |

TABLE S3 Comparison of epiphyllous species with epiphytic species on adjacent tree trunks at the first occurrence site (321 m).

| Habitat  Species | Epiphytic | Epiphyllous |
| --- | --- | --- |
| *Cololejeunea longifolia* | ✓ | ✓ |
| *Cololejeunea raduliloba* | ✓ | ✓ |
| *Cololejeunea spinosa* | ✓ | ✓ |
| *Cololejeunea japonica* |  | ✓ |
| *Frullania sp.* | ✓ |  |
| *Metzgeria furcata* | ✓ |  |
| *Metzgeria consanguinea* | ✓ |  |
| *Microlejeunea punctiformis* | ✓ | ✓ |
| *Acrolejeunea sandvicensis* | ✓ |  |
| *Lophocolea bidentata* | ✓ |  |
